# Supplementary material for: Hybrid Models and Biological Model Reduction with PyDSTool
Source: PLoS Comput Biol. 2012 Aug 9;8(8):e1002628. doi: 10.1371/journal.pcbi.1002628 (PMC3415397; doi:10.1371/journal.pcbi.1002628)
Supplement: Text S4 — Complete source code for the PyDSTool package (version 0.88.120504). Includes API documentation and help files linking to web pages. This file is identical to the current public release on Sourceforge.net. (ZIP) [file pcbi.1002628.s004.zip › PyDSTool/html/PyDSTool.Toolbox.phaseplane.plotter_2D-class.html]

xml version="1.0" encoding="ascii"?


PyDSTool.Toolbox.phaseplane.plotter\_2D


| Home | Trees | Indices | Help | PyDSTool |
| --- | --- | --- | --- | --- |

Package PyDSTool ::
Package Toolbox ::
Module phaseplane ::
Class plotter\_2D

[hide private]

[frames] | no frames]

# Class plotter\_2D

source code

```
object --+
         |
        plotter_2D
```

---

Plotting manager for phase plane analysis. A global instance of this
class called 'plotter' is already created and exported by this module.
Operate on that instance directly.

All plotting will take place in figure numbered self.curr\_fig, which
will be resized to the given shape and limits.

All added methods of form plot\_X take an optional named figure which
would correspond to whatever figure number is allocated in the
fig\_directory dictionary attribute. New plot methods of this kind must
first check that do\_display is True, and then call the 'setup' method
before, and 'teardown' after doing the necessary plotting.


|  |  |
| --- | --- |
| Instance Methods | [hide private] |

|  |  |
| --- | --- |
| \_\_init\_\_(self, x\_dom=None, y\_dom=None, figsize=`(`6`,` 5`)`, curr\_fig=1, do\_display=False)  x.\_\_init\_\_(...) initializes x; see x.\_\_class\_\_.\_\_doc\_\_ for signature | source code |

|  |  |
| --- | --- |
| set\_curr\_fig(self, name) | source code |

|  |  |
| --- | --- |
| setup(self, figname) | source code |

|  |  |
| --- | --- |
| teardown(self) | source code |

|  |  |
| --- | --- |
| plot\_nullcline(self, nullc, style, lw=1, N=100, marker=`'``'`, figname=None) | source code |

|  |  |
| --- | --- |
| plot\_vf(self, gen, xname, yname, col=`'``k``'`, N=20, subdomain=None, scale\_exp=0, figname=None)  Plot vector field in 2D | source code |

|  |  |
| --- | --- |
| plot\_fps(self, fps, coords=None, do\_evecs=False, markersize=10, figname=None)  Plot fixed point(s) in 2D | source code |

|  |  |
| --- | --- |
| plot\_line\_from\_points(self, A, B, style, lw=1, figname=None) | source code |

|  |  |
| --- | --- |
| plot\_point(self, A, style, figname=None) | source code |

|  |  |
| --- | --- |
| plot\_vline(self, x, style, figname=None) | source code |

|  |  |
| --- | --- |
| plot\_hline(self, y, style, figname=None) | source code |

**Inherited from `object`**:
`__delattr__`,
`__getattribute__`,
`__hash__`,
`__new__`,
`__reduce__`,
`__reduce_ex__`,
`__repr__`,
`__setattr__`,
`__str__`


|  |  |
| --- | --- |
| Properties | [hide private] |

**Inherited from `object`**:
`__class__`


|  |  |
| --- | --- |
| Method Details | [hide private] |

|  |  |
| --- | --- |
| \_\_init\_\_(self, x\_dom=None, y\_dom=None, figsize=`(`6`,` 5`)`, curr\_fig=1, do\_display=False)  *(Constructor)* | source code |

x.\_\_init\_\_(...) initializes x; see x.\_\_class\_\_.\_\_doc\_\_ for
signature

Overrides: object.\_\_init\_\_: *(inherited documentation)*

  


| Home | Trees | Indices | Help | PyDSTool |
| --- | --- | --- | --- | --- |

|  |  |
| --- | --- |
| Generated by Epydoc 3.0.1 on Fri May 4 15:24:10 2012 | http://epydoc.sourceforge.net |
